# Mathematical Modeling Approach and Simulation in Food Drying Applications

**DOI:** 10.3390/foods13030384

**Published:** 2024-01-24

**Authors:** Biljana Lončar, Lato Pezo

**Affiliations:** 1 Faculty of Technology Novi Sad, University of Novi Sad, Bulevar Cara Lazara 1, 21000 Novi Sad, Serbia; 2 Institute of General and Physical Chemistry, University of Belgrade, Studentski Trg. 12-16, 11000 Belgrade, Serbia

## 1. Introduction

Recent developments in the branch of food drying involve advancements in the development of mathematical models [1,2], spanning empirical, semi-empirical, and theoretical approaches [3,4]. Researchers have increasingly employed computational methods, including artificial neural networks [5,6], convolutional networks [7,8,9], random forests [10,11], support vector machines [12,13], and more, to analyze the impacts of diverse drying conditions and methods on food quality and safety. The integration of these computational techniques provides a sophisticated understanding of how different variables influence the drying process.

Furthermore, the ongoing exploration of mathematical modeling holds significant promise for enhancing food drying performance. Researchers are delving into the optimization of drying processes through the application of advanced mathematical models [14,15,16,17,18]. This approach not only contributes to a deeper comprehension of the underlying mechanisms but also offers practical insights into improving the efficiency and quality of food drying methods. As the field continues to evolve, the combination of mathematical modeling and computational methods is likely to assume a crucial role in shaping the future of food drying technologies.

**Figure 1:**
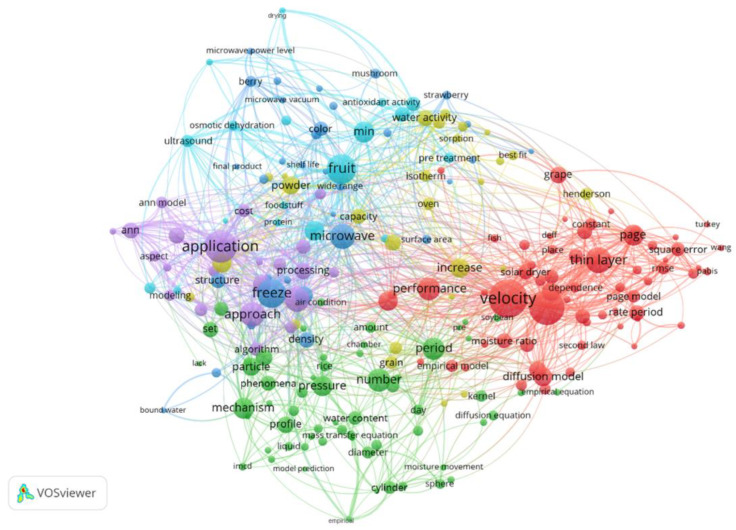
Co-occurrence analysis of the terms in “Mathematical Modeling Approach and Simulation in Food Drying Applications” research according to abstracts.

In order to detect patterns in the application of Mathematical Modeling Approaches and Simulations in the context of food drying, the VOSviewer program (ver. 1.6.20) was employed to visualize author and index keywords. For a comprehensive analysis, 178 searches were conducted of abstracts. As depicted in Figure 1, the exploration of Mathematical Modeling Approach and Simulation in Food Drying Applications, as per the abstracts, revealed distribution across six distinct clusters. Within the red cluster (associated with drying models), there was a compilation of diffusion models, with the terms “velocity”, “thin layer”, “Page”, and “performance” being the most frequently referenced. Additionally, less commonly occurring words, such as “diffusion model”, “second law”, and “rate period”, were also observed. The purple cluster gathered ANN models and applications in the food drying process. The terms most frequently occurring in the purple cluster corresponded to “application”, “approach”, “ANN”, and “processing”. In the light blue cluster, the most gentle drying techniques were displayed, including dominate words such as “fruit”, “min”, and “ultrasound”, while in the dark blue cluster, there were “microwave”, “freeze”, and “structure” (in which a slight decomposition of food structure was to be expected). The green cluster included empirical models applied in the food drying process. The terms that appeared most frequently in the green cluster were “period”, “mechanism”, and “mass transfer equation”. In the yellow cluster, the most frequently used words were “water activity”, “increase”, “power”, and “Hendreson”.

The size of the circle beneath each word indicates its frequency of occurrence. Various colors were utilized to illustrate separate clusters of closely related keywords, aiding in their categorization. The term cloud was visualized using the VOSviewer software, with data obtained from the Scopus database.

However, despite these advancements, a notable gap in knowledge persists. The existing research often lacks a comprehensive exploration of the complex interactions within food drying systems [19]. More specifically, there is a need for in-depth investigations into the nuanced effects of drying conditions on the quality and safety of different food products [20].

Additionally, gaps in the optimization of drying processes and the scalability of mathematical models for diverse food types remain areas that warrant further attention. Bridging these knowledge gaps holds the key to advancing the field and unlocking the full potential of mathematical modeling in optimizing food drying performance.

## 2. An Overview of Published Articles

This Special Issue encompasses a variety of studies covering the mathematical modeling approach and simulation in the context of food drying applications. It includes seven articles; the following paragraphs provide brief descriptions of each. It is important to note that the intention of this Editorial is not to delve into the details of each text but rather to interest readers to explore them independently. Furthermore, this Special Issue systematically addresses several critical gaps in the field of food drying through a diverse set of studies employing mathematical modeling approaches. The articles collectively contribute valuable insights and advancements, filling specific knowledge voids:

1. Dehydration of Chokeberries: Petković et al. investigated the dehydration of chokeberries through various methods, including microwave and microwave/convective dehydration. They observed rapid water loss during the initial stage of dehydration, irrespective of the drying method employed. The most effective conditions were found to be a microwave power of 900 W for 9 s and convective dehydration for 12 s. Dehydration time, energy consumption, and water holding capacity varied based on the method used. Microwave convective dehydration resulted in berries with enhanced freshness, acidity, and astringency, resembling fresh chokeberries, and a slight increase in crispness. This study offers valuable insights for the development of efficient drying methods for chokeberries.
2. Cassava Flour Optimization: Nainggolan et al. investigated the impact of drying parameters on moisture content and the whiteness index of cassava flour. They optimized parameters through response surface methodology and central composite design, examining microstructure alterations in cassava flour and assessing the outcomes. Temperature and drying time exhibited notable impacts on the moisture content and whiteness index of cassava flour. The constructed prediction models were accurate, identifying optimal conditions at 70 °C for 10 h. Validation results showed low relative deviations (0.12–1.48%). This study suggests potential research avenues, including cassava flour drying kinetics and exploring interactions between pretreatment and drying conditions to enhance quality.
3. Phenolic Preservation in P. Macrocarpa Fruits: Stephenus et al. studied the effects of oven-drying temperatures (40–80 °C) on phenolics, flavonoids, and antioxidant activity in P. macrocarpa fruits. They aimed to identify optimal drying conditions for high-quality raw material in food and nutraceutical production. The Midilli and Kucuk model best described the drying process. Higher temperature correlated with shorter drying time. Extraction yield was highest at 60 °C (33.99%). Total phenolic and flavonoid content peaked at 60 °C (55.39 mg GAE/g and 15.47 mg RE/g, respectively), with antioxidant activity at 84.49%. Bioactive components were effectively retained at 60 °C. Further research on industrial-scale extraction techniques and storage is recommended for commercialization in foods and nutraceuticals.
4. Osmotic Dehydration of Pork Meat Proteins: Ostojić et al. investigated the kinetic and thermal properties of fresh and osmotically dehydrated pork meat proteins (*Longissimus dorsi*). They applied isoconversional differential Friedman and integral Ortega methods to scrutinize kinetic data. The objective of this study was to thermally characterize and contrast the denaturation of proteins in fresh and dried meat using differential scanning calorimetry (DSC). Osmotic dehydration with sugar beet molasses led to thermal stabilization, resulting in dried meat proteins existing in a partially unfolded state. While thermally stabilized, osmotically dried proteins were found to be less stable kinetically and thermodynamically than fresh meat proteins. The study also suggested the potential role of shear stress in protein denaturation within the formed protein matrix. DSC was deemed effective for understanding the complex processes during osmotic dehydration. Additional research is suggested to gain a complete understanding of the denaturation process of meat proteins dried in molasses.
5. Utilization of Ultrasonic Technology in Seafood Drying: Fikry et al. investigated the use of ultrasonic technology in the drying process of Asian seabass (Lates calcarifer) fish skin under various convection drying temperatures (45, 55, and 65 °C), both with and without ultrasound pre-treatment. Ultrasound pretreatment significantly decreased drying time, with dried samples confirmed as microbiologically safe. The modified Page model satisfactorily described drying behavior, and ultrasound-treated samples exhibited higher effective diffusivity coefficients, indicating improved moisture removal. Ultrasound pre-treatment resulted in a 22% average reduction in specific energy consumption and enhanced energy efficiency. The dried samples subjected to ultrasound pre-treatment exhibited a more porous and open structure. Overall, this study suggests that ultrasound pretreatment accelerates drying, reduces energy consumption, and enhances the efficiency and sustainability of seafood drying processes. These findings have implications for the seafood industry and contribute valuable insights to enhance food-processing operations.
6. Advancing Fruit Drying Efficiency with an Innovative Vacuum Dryer: Šumić et al. endeavored to improve the efficiency of fruit drying through the development and experimentation of an innovative vacuum dryer featuring an ejector system. The prototype, evaluated on sour cherries and apricots, exhibited comparable results in terms of moisture content, aw value, phenol, flavonoid, anthocyanin content, and antioxidant activity (FRAP, DPPH, ABTS) compared to a conventional vacuum dryer equipped with a vacuum pump. The innovative dryer proved economically advantageous due to lower investment and maintenance costs. Quality criteria were met in both dryers, with the ejector system dryer standing out economically. This research concluded that the innovative vacuum dryer is a viable and cost-effective option for fruit drying, with comparable energy efficiency to a vacuum pump.
7. Drying Kinetics and Quality of Rila Tomato Peels: Popescu et al. aimed to develop drying kinetic models for Rila tomato peels and predict optimal drying temperature while considering the impact on food quality. Six temperatures (50–75 °C) were studied, maintaining a consistent final moisture. Various mathematical models based on Fick’s second law of diffusion were employed to predict kinetic parameters, with a particular emphasis on statistical validation. Drying temperature significantly affected moisture removal, final product quality, and energy consumption. Carotenoid degradation, specifically lycopene and β-carotene, increased with higher temperatures. The two-term model accurately represented tomato peel drying behavior. Two degradation models were formulated for carotenoids, showing significant degradation at higher temperatures. The recommended drying temperature for Rila tomato peels to preserve carotenoids and achieve energy efficiency is 50 °C.
8. ANN Optimization in Sweet Potato Varieties Drying Processes: The study performed by Šovljanski et al. delves into harnessing the untapped potential of artificial neural network (ANN) optimization to enhance diverse drying methods and their impact on the characteristics of different sweet potato varieties. Investigating the intricate interplay between drying techniques and the distinctive traits of white, pink, orange, and purple sweet potatoes, this experimental study highlights the influence of ANN-driven optimization on various food-related attributes, including color, phenols content, and biological activities (antioxidant, antimicrobial, anti-hyperglycemic, and anti-inflammatory), as well as chemical and mineral contents. The findings reveal substantial variations in the effectiveness of drying methods for different sweet potato types, emphasizing the necessity for tailored optimization strategies. Purple sweet potatoes particularly stand out as robust carriers of phenolic compounds, demonstrating superior antioxidant activities. This study also discloses optimized parameters for dried sweet potatoes, such as a total phenols content of 1677.76 mg/100 g, anti-inflammatory activity of 8.93%, and anti-hyperglycemic activity of 24.42%. Enhanced antioxidant capability is demonstrated through DPPH•, ABTS•+, RP, and SoA assays, with values of 1500.56, 10,083.37, 3130.81, and 22,753.97 μg TE/100 g, respectively. Additionally, the lyophilized sample achieves a minimum moisture content of 2.97%, maintaining favorable chemical and mineral contents.

Collectively, these studies contribute to bridging gaps in knowledge related to the efficiency, quality, and sustainability of food drying processes, offering practical implications for both research and industry practices.

## 3. Conclusions

This Special Issue sets the stage for future research avenues. The studies provide a foundation for the further exploration of and improvement in food drying processes. While the studies outlined here offer valuable insights into individual aspects of food drying, future research should delve into a more comprehensive understanding of the intricate interactions between various parameters. This includes exploring the synergistic effects of different drying conditions on multiple quality attributes. 

The scalability of mathematical models across different food types and processing scales remains an important consideration. Future research should aim to develop models that are adaptable to diverse food products and can be applied on an industrial scale, ensuring practical relevance and applicability.

Emerging technologies, such as innovative vacuum dryers and ultrasound pretreatment, show promise in enhancing drying efficiency and quality. Future research should continue to explore and refine these technologies, considering their integration into large-scale food processing operations.

The sustainability of food drying processes is a critical concern. Future studies should focus on optimizing drying methods not only for efficiency but also for reduced energy consumption and environmental impact. This involves exploring eco-friendly drying techniques and assessing the overall sustainability of the drying process.

Understanding the mechanisms behind the preservation of key quality attributes, such as phenolics, flavonoids, and carotenoids, during drying is essential. Future research should investigate the underlying biochemical and physical processes used to develop targeted strategies for preserving nutritional and sensory qualities.

Collaborative efforts between food scientists, engineers, and technologists are essential for advancing this field. Future research should embrace a multi-disciplinary approach, combining expertise in mathematics, physics, biology, and engineering to provide holistic insights into the complex dynamics of food drying.

As the food industry evolves, understanding consumer preferences and market trends becomes crucial. Future research should incorporate market-driven considerations, exploring how drying processes can align with consumer demands for convenience, nutrition, and sustainable practices.

In conclusion, while this Special Issue has made significant strides in addressing gaps in the field of food drying, it also serves as a springboard for future research endeavors. By embracing these primary focuses, researchers can contribute to the continued enhancement of food drying technologies, ensuring both efficiency and sustainability in the ever-evolving landscape of food science and technology.

## Data Availability

No applicable.

## References

[1] Sun Q., Zhang M., Mujumdar A.S. (2019). Recent developments of artificial intelligence in drying of fresh food: A review. Crit. Rev. Food Sci. Nutr..

[2] Chen C., Pan Z. (2023). An overview of progress, challenges, needs and trends in mathematical modeling approaches in food drying. Dry. Technol..

[3] Zhang W., Ke W., Chang C. (2022). Artificial Neural Network Assisted Multiobjective Optimization of Postharvest Blanching and Drying of Blueberries. Foods.

[4] Li J., Deng Y., Xu W., Zhao R., Chen T., Wang M., Xu E., Zhou J., Wang W., Liu D. (2023). Multiscale modeling of food thermal processing for insight, comprehension, and utilization of heat and mass transfer: A state-of-the-art review. Trends Food Sci. Technol..

[5] Demir H., Demir H., Lončar B., Pezo L., Brandić I., Voća N., Yilmaz F. (2023). Optimization of Caper Drying using Response Surface Methodology and Artificial Neural Networks for Energy Efficiency Characteristics. Energies.

[6] Zadhossein S., Abbaspour-Gilandeh Y., Kaveh M., Szymanek M., Khalife E.D., Samuel O., Amiri M., Dziwulski J. (2021). Exergy and energy analyses of microwave dryer for cantaloupe slice and prediction of thermodynamic parameters using ANN and ANFIS algorithms. Energies.

[7] Iheonye A.C., Raghavan V., Ferrie F.P., Orsat V., Gariepy Y. (2023). Monitoring Visual Properties of Food in Real Time During Food. Food Eng. Rev..

[8] Przybył K., Koszela K. (2023). Applications MLP and other methods in artificial intelligence of fruit and vegetable in convective and spray drying. Appl. Sci..

[9] Geng L., Xu W., Zhang F., Xiao Z., Liu Y. (2018). Dried jujube classification based on a double branch deep fusion convolution neural network. Food Sci. Technol. Res..

[10] Kaveh M., Çetin N., Khalife E., Abbaspour-Gilandeh Y., Sabouri M., Sharifian F. (2023). Machine learning approaches for estimating apricot drying characteristics in various advanced and conventional dryers. J. Food Process Eng..

[11] Khanchi A., Birrell S., Mitchell R.B. (2018). Modelling the influence of crop density and weather conditions on field drying characteristics of switchgrass and maize stover using random forest. Biosyst. Eng..

[12] Das M., Akpinar E.K. (2018). Investigation of pear drying performance by different methods and regression of convective heat transfer coefficient with support vector machine. Appl. Sci..

[13] Hadjout-Krimat L., Belbahi A., Dahmoune F., Hentabli M., Boudria A., Achat S., Remini H., Oukhmanou-Bensidhoum S., Spigno G., Madani K. (2023). Study of microwave and convective drying kinetics of pea pods (*Pisum sativum* L.): A new modeling approach using support vector regression methods optimized by dragonfly algorithm techniques. J. Food Process Eng..

[14] Sun Y., Zhang M., Mujumdar A. (2019). Berry drying: Mechanism, pretreatment, drying technology, nutrient preservation, and mathematical models. Food Eng. Rev..

[15] Filipović V.S., Filipović J.S., Petković M.M., Filipović I.B., Miletić N.M., Đurović I.B., Lukyanov A.D. (2022). Modeling convective thin-layer drying of carrot slices and quality parameters. Therm. Sci..

[16] Akter F., Muhury R., Sultana A., Deb U.K. (2022). A comprehensive review of mathematical modeling for drying processes of fruits and vegetables. Int. J. Food Sci..

[17] Inyang U.E., Oboh I.O., Etuk B.R. (2018). Kinetic models for drying techniques—Food materials. Adv. Chem. Eng. Sci..

[18] Castro A.M., Mayorga E.Y., Moreno F.L. (2018). Mathematical modelling of convective drying of fruits: A review. J. Food Eng..

[19] Onwude D.I., Hashim N., Janius R.B., Nawi N.M., Abdan K. (2016). Modeling the thin-layer drying of fruits and vegetables: A review. Compr. Rev. Food Sci. Food Saf..

[20] Depiver J.A., Mallik S. (2023). An empirical study on convective drying of ginger rhizomes leveraging environmental stress chambers and linear heat conduction methodology. Agriculture.

